# Italian regional variation in amenable mortality 2015/2019: impact of outsourcing, expenditures and socio-economic variables

**DOI:** 10.3389/fpubh.2026.1832576

**Published:** 2026-05-14

**Authors:** Francesco Conrado, Ettore Minutiello, Jacopo Lenzi, Pietro Marraffa, Maria Michela Gianino

**Affiliations:** 1 Department of Sciences of Public Health and Pediatrics, University of Turin, Turin, Italy; 2 Local Health Authority City of Turin, Maria Vittoria-Amedeo di Savoia Hospital, Turin, Italy; 3 Department of Biomedical and Neuromotor Sciences, University of Bologna, Bologna, Italy

**Keywords:** amenable mortality, health inequities, health system performance, healthcare outsourcing, socioeconomic factors

## Abstract

**Background:**

This study addresses the need to monitor regional performance in decentralized health systems and determine the main variables associated with regional variability. Amenable mortality serves as a pivotal performance indicator of healthcare systems.

**Methods:**

An observational study was grounded from 2015 to 2019. The regional variability in amenable mortality, using age-standardized death rates (SDRs), was analyzed. Variation among regions was examined against other variables; Socio-economic (net household income, poverty rates, education level, and ethnic minority rates), Healthcare expenditure variables (per capita public and private health expenditure, inpatient and outpatient services outsourcing toward for-profit providers). A multivariate regression model was utilized, including all variables to examine the overall associations with amenable mortality.

**Results:**

The results showed that from 2015 to 2019 the SDRs range widely among regions. We found in the multivariate regression model that an annual increase of one percentage point of outsourcing to the private sector, poverty rate, ethnic minority rate were associated with an annual increase in amenable mortality; by contrast, an annual increase of one percentage point of household net income was associated with a slight annual decrease in amenable mortality.

**Conclusion:**

The study highlights that in decentralized systems, performance, measuring in amenable mortality, can be different. Some variables can explain and affect this variability. Policy makers should make an integrated reading of these findings to undertake measures capable of improving regional amenable mortality and should monitor healthcare system metrics, especially in the face of evolving practices such as outsourcing.

## Introduction

Amenable mortality is defined as causes of death that are largely preventable by fast and effective healthcare interventions to reduce the number of deaths (1). Essentially, they are the deaths that could be sidestepped with timely medical interventions, encompassing both secondary prevention and treatments post-disease onset. This has been termed “treatable mortality”, a nomenclature emphasizing its connection with judicious healthcare (2).

Effective healthcare manifests when it can mitigate the risk of death from an array of causes via preventative and therapeutic actions (3).

Rutstein and colleagues first proposed the concept, along with a list of conditions that are treatable or preventable based on the prevailing medical knowledge and technology (4). Thereafter, amenable mortality has been suggested as a potential performance indicator of the healthcare system by Nolte and McKee (5, 6) and Tobias and Yeh (7). Since being introduced, variations in amenable mortality, either over time or between different geographical areas (8–11) have been explored and its associated explanatory factors have been investigated. Studies have demonstrated that many different factors contribute to reduce the amenable mortality, including level of education (12), income (13), socioeconomic status (14, 15) and healthcare expenditure (16, 17) and the relationship has been found in studies across European and OECD countries (14, 18, 19).

In the Italian context, a growing body of literature has examined the effects of fiscal constraints and healthcare system organization on health outcomes. Arcà et al. (2020) used a difference-in-differences design to show that cost containment measures imposed on regions under recovery plans (Piani di Rientro) led to increases in avoidable mortality (20) Bordignon et al. analyzed hardening budget constraints through administrative subordination in regional health services (21). Depalo examined the side effects of recovery plans on health outcomes using a nonparametric bounding approach (22). Cirulli and Marini assessed whether austerity measures were associated with health distress, providing evidence from the Italian setting (23). Golinelli et al. analyzed the relationship between health expenditure and all-cause mortality across Italian regions over a 15-year period using panel data methods (24). While these studies have primarily focused on the effects of fiscal austerity and cost containment, to our knowledge no study has specifically examined the role of healthcare outsourcing to for-profit providers as a determinant of amenable mortality in Italy. Our study addresses this gap by jointly modeling outsourcing alongside socioeconomic determinants, thereby capturing a distinct organizational dimension of healthcare delivery.

Instead, little is known about territorial variation in amenable mortality rates (19, 25–27). The Italian context is an interesting case on focus on. The Italian Health System has been interested in the federalist process since 1992. Legislative measures, Decree Law 502/92, Constitutional Law. 3/2001, Decree Law 56 of 2,000, have created a healthcare system organized at national, regional and local level and have provided assignment of responsibilities and resources among different levels. Health protection is a shared responsibility between the State and regions, with the former establishing the fundamental principles and objectives of the health system, delineates essential care levels (Livelli Essenziali di Assistenza, LEA), a repertoire of publicly financed health services accessible regionally (28). Regional authorities retain discretion in organizational strategies for LEA provision (29). The local level is responsible for ensuring healthcare services for the target population, both directly through its own facilities and through outsourcing. Given this decentralized setup, vigilantly monitoring regional performance becomes paramount and determining the main factors associated with regional variation in amenable mortality, may play a role in the design of public health policy and prevention.

Addressing this gap our study aims to:

1. Dissect the variability in amenable mortality rates from 2015 to 2019 across Italian regions;
2. Investigate how key regional socio-economic indicators are associated with amenable mortality rates;
3. Examine whether more healthcare expenditure variables, including outsourcing, are associated with regional amenable mortality rates.

## Materials and method

This observational study was grounded in secondary data spanning from 2015 to 2019. This particular timeframe was selected for two critical reasons. First, we aimed to eschew any potential influences or distortions stemming from the COVID-19 pandemic on health outcomes and health financial resources. Second, as of our study's commencement, the certified balance sheets available from the Economic-Financial Regional Database Repository of the Ministry of Health were definitive only up to the year 2020, thus limiting the latest possible data year to 2019 to ensure accuracy and completeness.

Data are retrieved from the Italian National Institute of Statistics (ISTAT), where the causes of death are coded using the ICD-10 classification. The amenable mortality rate (SDRs) is calculated based on the Organization for Economic Co-operation and Development's (OECD) list of pathologies for each region in Italy (2). We computed the amenable mortality rate as the mean yearly number of deaths per 100,000 inhabitants aged 0–74 years, with stratification conducted according to region, including 19 regions and the two autonomous provinces of Trentino and Alto Adige. To account for variations in age distribution across these regions, we employed age-standardized death rates (SDRs), utilizing the 2011 Italian population as the standard reference population. The linchpin variable for this analysis comprised diseases deemed responsive to healthcare intervention, as meticulously outlined in the OECD pathology list (2).

Socio-economic data, include net household income, poverty rates, education level, and ethnic minority rates, and were obtained from ISTAT public datasets. These are defined as follows:

- Net household income: defined as the “income available to households, such as wages and salaries, income from self-employment and unincorporated enterprises, income from pensions or other social benefits, and income from financial investments (less any payments of tax, social insurance contributions and interest on financial liabilities)” (30).
- Poverty rates: defined based on the use of a poverty threshold termed as the International Standard of Poverty Line (ISPL). This standard designates a two-member household as impoverished if its consumption expenditure is less than or equal to the average per-capita consumption expenditure.
- Educational levels: presented as the percentage of people per region holding primary school certificates, middle school completion, technical school diplomas, high school diplomas, university degrees, or beyond.
- Ethnic minority rates: defined as the percentage of people per region holding residence permits for foreign citizens.

Healthcare expenditure variables, such as per capita public health expenditure and per capita private health expenditure, were extracted from the health observatory source Osservasalute (31). The per capita public health expenditure provides a measure of the average monetary resources utilized for each individual in a specific region to address the provision of healthcare services over a reference period. Conversely, the per capita private health expenditure represents the financial burden directly borne by each citizen for out-of-pocket payments, service fees, and voluntary contributions.

Regarding the outsourcing details, data were drawn from the certified balance sheet reports available at the Economic-Financial Regional Database Archive of the Ministry of Health. We retrieved the expenditure for outpatient and inpatient (respectively codes BA0530 and BA0840) outsourcing toward for-profit providers. Subsequently, to provide a clearer perspective on the extent of outsourcing within regions, we determined the following ratios:

- Inpatient outsourcing ratio: this measures the proportion of healthcare services for hospital care purchased from private for-profit providers relative to the overall acquisitions for hospital assistance within the region.
- Outpatient outsourcing ratio: this evaluates the proportion of ambulatory care services sourced from private for-profit providers to the total ambulatory care services acquired by regional health authorities.

These variables were also included because outsourcing in the healthcare sector represents a strategic shift wherein public entities delegate specific services to third party providers, often from the private sector. This move is primarily motivated by potential cost savings and increased efficiency. However, integrating market-based mechanisms in a traditionally public domain raises critical questions about the quality of care and patient outcomes.

### Statistical analysis

We engaged in a thorough descriptive analysis of amenable mortality rates for each region. These rates were graphically illustrated using a forest plot, which facilitated the visual representation of the central estimate alongside its 95% confidence intervals (C.I.) for each year in focus. Furthermore, by computing the mean amenable mortality rates for all regions over the entirety of our study duration, we garnered a consolidated perspective of the mortality trends. To determine if there are statistically significant differences between regions regarding amenable mortality rates, the Kruskal- Wallis rank sum test was used.

To assess the relationship between amenable mortality and the various socio-economic and healthcare expenditures variables, both simple and multiple linear regression models were employed. Each variable was independently tested in the simple linear regression model. Subsequently, a multivariate regression model was utilized, including all variables to examine the overall associations with amenable mortality.

Formally, the bivariate regression models (Table 1) are specified as: SDR_it = α + βX_it + ε_it, where SDR_it denotes the age-standardized amenable mortality rate for region i in year t, X_it is the explanatory variable, and ε_it is the error term. The multivariate regression model (Table 2) is specified as: SDR_it = α + β1·Outsourcing_it + β2·PubExp_it + β3·PrivExp_it + β4·Income_it + β5·Poverty_it + β6·Ethnic_it + β7·Education_it + ε_it.

**Table 1:** Linear regression analyses between amenable mortality rates and socioeconomic and healthcare expenditures variables.

Variables	Intercept estimate	Coefficient estimate	Std. error	*t*-value	*p*-value	Adjusted R^2^
Socioeconomic variable						
Low educational level	34·44	1·77	0·25	7·22	<0·001	0·33
Poverty rate	55·35	0·91	0·089	10·24	<0·001	0·51
Household net income	1·22e + 02	−1·80e-03	1·27e−04	−14·11	<0·001	0·66
Ethnic minority rate	78·84	−2·23	0·29	−7·80	<0·001	0·36
Healthcare expenditure variables						
Public health expenditure (per capita)	119·22	−0·027	0·0052	−5·29	<0·001	0·21
Private health expenditure (per capita)	90·68	−0·039	0·0053	−7·32	<0·001	0·34
Inpatient outsourcing	60·66	0·12	0·049	2·38	0·020	0·043
Outpatient outsourcing	62·06	0·081	0·050	1·63	0·11	0·016
Outsourcing (in and outpatient)	60·40	0·12	0·052	2·28	0·025	0·039

**Table 2:** Multivariate regression model.

Predictor	Estimate	Std. error	C.I. 95%	*t*-value	*p*-value
(Intercept)	139·90	15·46	(109·44, 170·36)	9·05	<0·001
Outsourcing	0·089	0·031	(0·03, 0·15)	2·85	0·0055
Public health expenditure (per capita)	−0·0049	0·0047	(−0·01, 0·01)	−1·04	0·30
Private health expenditure (per capita)	0·0039	0·0051	(−0·01, 0·02)	0·77	0·44
Household net income	−0·0024	0·0033	(−0·01, −0·00)	−7·35	<0·001
Poverty rate	0·35	0·14	(0·07, 0·63)	2·49	0·014
Ethnic minority rate	1·36	0·44	(0·48, 2·24)	3·05	0·0030
Low educational level	−0·48	0·28	(−1·04, 0·08)	−1·74	0·085

Residual standard error: 5·089 on 91 degrees of freedom (6 observations deleted due to missingness), Multiple R-squared: 0·75, Adjusted R-squared: 0·73, F-statistic: 38·8 on 7 and 91 DF, *p*-value: < 2·2e−16. The statistical analysis met the assumptions for the application of linear regression, as indicated by the distribution of residuals, and provided a robust examination of the variables' influences on amenable mortality rates.

Our dataset has a panel structure (21 territorial units observed over 5 years, yielding 105 region-year observations). We adopted a pooled ordinary least squares (OLS) approach rather than fixed-effects or random-effects panel models for the following reasons: (1) the primary aim of this study is to describe cross-sectional associations between regional characteristics and amenable mortality, rather than to estimate within-region causal effects over time; (2) several key explanatory variables (e.g., outsourcing ratios, poverty rates) exhibit limited within-region temporal variation over the short five-year window, which would severely limit the statistical power of fixed-effects models; (3) fixed-effects estimation would absorb precisely the between-region variation that is the focus of our analysis. To account for within-region serial correlation, standard errors are clustered at the regional level. We acknowledge that the pooled OLS approach does not control for unobserved time-invariant regional heterogeneity, and therefore our results should be interpreted as associations rather than causal effects. Six observations were deleted due to missingness in some variables for specific region-years.

All statistical analyses were conducted using RStudio version 2023.03.0+386. The level of statistical significance was set at a *p*-value of 0·05.

## Results

From 2015 to 2019 the mean SDR in Italy was 66·2 per 100,000 inhabitants, ranging from 50·6 per 100,000 inhabitants in Provincia Autonoma di Trento to 88·0 per 100,000 inhabitants in Campania. Results indicated that seven regions (Lombardia, Emilia Romagna, Trentino Alto-Adige, Veneto, Umbria, Marche) had statistically significant SDRs lower than the national average while for nine regions (Valle d'Aosta, Piemonte, Liguria, Friuli Venezia-Giulia, Abruzzo, Molise, Puglia, Basilicata, Sardegna) the SDRs did not differ statistically from the national average. Lastly, four regions (Lazio, Campania, Calabria, Sicilia) showed SDRs statistically significantly higher than the national average.

**Figure 1:**
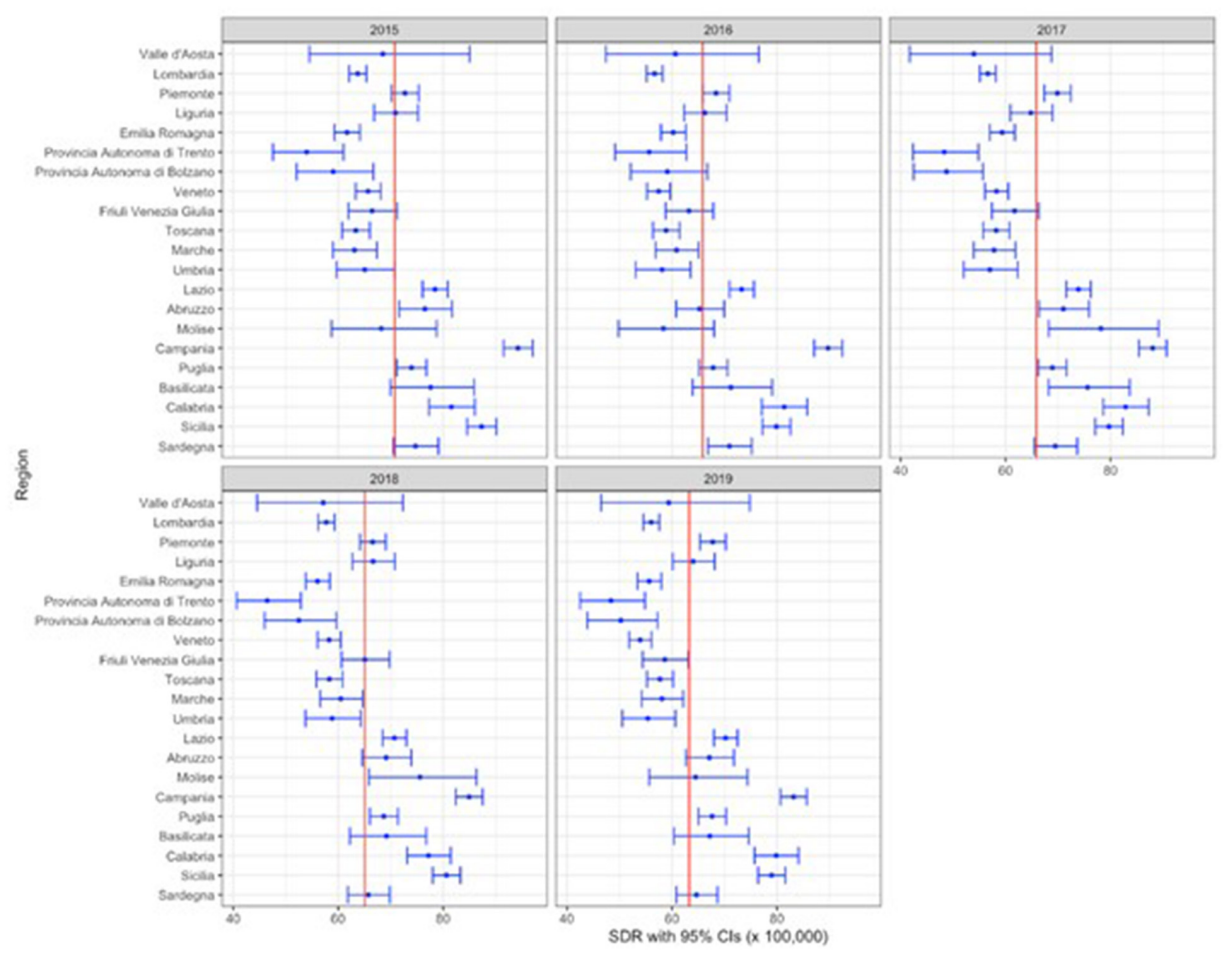
Forest plot of SDRs (C.I. 95%) across Italian regions between 2015 and 2019.

Figure 1 shows amenable mortality across Italian regions from 2015 to 2019 and provides a clear depiction of point estimates and their corresponding confidence intervals. Kruskal-Wallis chi-squared value was 88·75 (df = 20) with *p* value < 0·001, indicating that there is strong evidence of a difference in the amenable mortality rates variable across Italian regions.

The linear regression analyses between amenable mortality rates and socioeconomic variables are shown in Table 1. A significant inverse relationship was observed between household net income and amenable mortality rates (*p* < 0·001).

Higher rates of poverty and lower levels of education were associated with higher amenable mortality rates (respectively *p* < 0·001; *p* < 0·001). The ethnic minority rate was inversely related to amenable mortality (*p* < 0.001).

The linear regression analyses between amenable mortality rates and healthcare expenditure variables revealed an inverse association between both public and private health expenditure (per capita) and amenable mortality rates (respectively *p* < 0.001; *p* < 0·001). The rate of inpatient outsourcing was positively associated with amenable mortality (*p* = 0·019). A similar positive association was observed when both inpatient and outpatient outsourcing were considered together (*p* = 0·025). Outpatient outsourcing alone, however, did not show a significant relationship (*p* = 0·11).

It is important to note that Table 1 reports bivariate (unadjusted) regressions, where each explanatory variable is tested separately; therefore, coefficients capture unconditional correlations between each variable and amenable mortality. Table 2, in contrast, reports the multivariate (adjusted) regression in which all variables are included simultaneously, and coefficients represent partial correlations—i.e., the association of each variable with mortality holding all other variables constant. Differences between the two tables may reflect confounding, multicollinearity, or suppression effects. The multivariate model (Table 2) is the relevant model for substantive interpretation.

In the multivariate regression model, household net income, poverty rate, and ethnic minority rate maintained significant associations with amenable mortality rates (*p* < 0·001; *p* = 0·015; *p* = 0·003 respectively). The influence of education level, public health expenditure (per capita), and private health expenditure (per capita) were not statistically significant in this model (*p* = 0·085; *p* = 0·30; *p* = 0·44, respectively). Outsourcing remained significantly related to amenable mortality (*p* = 0·005). This model accounted for approximately 73% of the variation in amenable mortality rates, indicating a strong fit to the data (Table 2).

## Discussion

This study aims to examine the variation of amenable mortality rates across Italian regions from 2015 to 2019, explore the association between amenable mortality and socioeconomic variables of the regional population, and describe the correlation between the amenable mortality and the regional healthcare expenditures levels, and the outsourcing toward for-profit healthcare sector during the same period.

Our results showed that from 2015 to 2019 the SDRs range widely among regions. We found in the multivariate regression model that an annual increase of one percentage point of outsourcing to the private sector, poverty rate, ethnic minority rate were associated with an annual increase in amenable mortality; by contrast, an annual increase of one percentage point of household net income was associated with a slight annual decrease in amenable mortality.

The delineated regional disparities in SDRs and the stark contrast between northern and southern regions mirrors previous studies results about the uneven distribution of health outcomes in Italy. (27, 32).

Our findings suggest that the delineated regional disparities in SDRs might be attributed to some of the socioeconomic and expenditure variables.

Consistently, the household net income and poverty rate showcase a significant negative and positive, respectively, association with amenable mortality, while, unexpectedly, the educational does not report a significant association. Nonetheless, these findings should be read in an integrated manner. Higher socioeconomic status not only leads to higher availability, but is often intertwined with higher education levels, factors that ensure better access to medical services, (33–35) and also increased health knowledge, better health behaviors improving amenable mortality (36, 37). Conversely, High poverty levels can lead to reduced access to healthcare services, lower health literacy, and increased susceptibility to diseases due to poor living conditions (38, 39).

Among the statistically significant predictors, ‘Outsourcing' is particularly noteworthy. According to our model, a one percentage point increase in the outsourcing ratio corresponds to an uptick standardized death ratio by 0·089 (*p*-value = 0·0055). This finding aligns with the discourse surrounding potential quality disparities in outsourced healthcare. For example, Lafortune and colleagues previously illuminated the potential pitfalls of fragmented care when services are outsourced, emphasizing the gaps it can create in the continuity of care (40). Continuity, a pivotal component in healthcare quality, ensures that patients receive coherent and linked services, avoiding preventable complications. This continuum may be disrupted in outsourced models, particularly if the outsource partners don't share integrated information systems or if the standard care protocols differ markedly. Contrasting our results with the broader literature on amenable mortality reveals that amenable deaths often arise due to systemic issues rather than individual patient comorbidities. For instance, a study by James highlights that lapses in coordination, misdiagnoses, and failure to adhere to best practices often underpin these regrettable outcomes (41).

Potential downsides related to patient outcomes, also might be due when considering that outsourced services might sometimes prioritize cost-cutting measures, which can inadvertently compromise the quality of care. Our results seem to echo the concerns raised by Goodair and colleagues in their study on primary care in Europe, where they illuminated a correlation wherein a one percentage point annual uptick in for-profit outsourcing was associated with a 0.38% annual rise in treatable mortality or an additional 0.29 deaths per 100,000 populace the subsequent year (28). Given the gravity of the potential repercussions, such as increased treatable mortality, it is imperative for policymakers to rigorously evaluate and monitor outsourced services. Ensuring that these services align with the broader healthcare objectives can not only optimize the benefits of outsourcing but also mitigate its potential risks.

To contextualize the economic significance of the estimated coefficients, it is useful to relate them to the mean SDR of 66.2 per 100,000 inhabitants. The outsourcing coefficient of 0.089 implies that a one-percentage-point increase in the outsourcing ratio is associated with an increase of 0.089 deaths per 100,000 inhabitants, corresponding to approximately 0.13% of the mean SDR. Over the observed inter-regional range of outsourcing ratios (spanning approximately 30 percentage points), this translates into a predicted difference of approximately 2.7 deaths per 100,000—a non-negligible magnitude. By comparison, Goodair et al. (28) reported that a one-percentage-point increase in for-profit outsourcing in England was associated with 0.29 additional deaths per 100,000 in treatable mortality. The poverty rate coefficient of 0.35 indicates that a one-percentage-point increase is associated with an increase of 0.35 deaths per 100,000 (approximately 0.53% of the mean SDR). For household net income, a €1,000 increase is associated with a decrease of approximately 2.4 deaths per 100,000 (3.6% of the mean SDR), underscoring the substantial role of socioeconomic conditions. The ethnic minority rate coefficient of 1.36 deaths per 100,000 per percentage point (approximately 2.1% of the mean SDR) likely reflects barriers to healthcare access faced by migrant populations, including language barriers, legal status, and cultural factors.

Notably, the sign of the ethnic minority rate coefficient reverses between the bivariate model (Table 1: β = −2.23, *p* < 0.001) and the multivariate model (Table 2: β = 1.36, *p* = 0.003). This is a classic example of Simpson's paradox, arising from omitted variable bias in the bivariate setting. In the unadjusted regression, the negative coefficient likely reflects confounding: regions with higher proportions of foreign residents tend to be wealthier northern regions with overall better health outcomes and healthcare systems. Once income, poverty, and other variables are controlled for in the multivariate model, the partial effect of the ethnic minority rate becomes positive, possibly capturing barriers to healthcare access experienced by migrant populations, which is consistent with the literature on migrant health disparities (38).

Both public and private per capita health expenditure did not achieve statistical significance in the multivariate model, despite showing significant bivariate associations (Table 1). Several explanations may account for this finding. First, multicollinearity is likely: health expenditure is strongly correlated with household income and other socioeconomic variables, as wealthier regions tend to allocate more resources to healthcare. When these correlated predictors are included simultaneously, the independent contribution of expenditure may be absorbed. Second, it is possible that how resources are allocated and organized—including aspects of care coordination, service quality, and governance—matters more for amenable mortality than the aggregate amount spent. Third, the limited within-region variation in expenditure over the short study period may reduce the ability to detect its effects. Importantly, this non-significance should not be interpreted as evidence that healthcare resources do not matter for health outcomes, but rather that, in the context of our model and data, expenditure levels alone do not capture the efficiency, equity, or quality of healthcare delivery (42–44).

In summary, while financial resources remain essential for healthcare delivery, our findings suggest that in the Italian context, organizational factors such as outsourcing and socioeconomic conditions—including household income and poverty rate—may play a more directly measurable role in explaining regional variation in amenable mortality when modeled alongside aggregate expenditure levels.

This study presents several limitations and strengths.

Our analysis draws primarily from secondary data sources, which inherently pose challenges in inferring direct causal relationships, even though meaningful associations can be ascertained. In evaluating the effects of healthcare outsourcing, we employed an aggregated approach to the outsourcing expense ratio. This method, while offering an overarching perspective, may mask the diversity and specificity of services outsourced by individual regions. Such nuances can impede our ability to discern the impact of distinct outsourced services on amenable mortality rates.

Furthermore, omitted variable bias may affect our estimates. Important variables not included in our model—such as physician density, hospital bed supply, diagnostic infrastructure, prevalence of chronic diseases, behavioral risk factors, and urban-rural population structure—may be correlated with both our explanatory variables and amenable mortality. For instance, regions with weaker healthcare infrastructure may simultaneously resort more to outsourcing and experience higher mortality, potentially leading to an overestimation of the outsourcing effect. Similarly, regions with higher chronic disease prevalence may have both higher mortality and different socioeconomic profiles. The absence of these covariates means that the estimated coefficients should be interpreted with caution and as partial associations, not causal effects. Future studies should aim to incorporate these variables as data availability improves.

Additionally, the use of pooled OLS rather than panel data methods (e.g., fixed-effects or random-effects models) constitutes a methodological limitation. While justified by the short time span and the cross-sectional focus of our analysis, the pooled approach does not control for unobserved time-invariant regional characteristics that may confound the estimated associations. Potential endogeneity of outsourcing—for instance, if regions with worse health outcomes are more likely to outsource as a cost-saving strategy—remains a concern that our observational design cannot fully address.

Additionally, while we utilized amenable mortality as a representative metric to gauge healthcare system performance, it's important to underscore that it is one among various potential indicators. Our choice is informed by its prevalence in literature and its recognition by prominent organizations like the OECD, yet it captures a specific dimension of the broader landscape of healthcare outcomes. Finally, with an adjusted R-squared of 0·73, the model accounts for nearly 73% of the variance in amenable mortality, indicating its strong explanatory power. Yet, the unexplained 27% variance underscores the presence of other potential confounding variables or external factors not encompassed in our model, such as lifestyle choices, disease prevalence, or health system metrics.

First strength is that our analysis was performed disaggregated data by regional socioeconomic characteristics and territorial expenditure levels, bringing to light variations within the country and among regions. Evidence from Finland and Poland, for example, pointed that in analysis performed only at country level potentially large variations within regions and populations in performances of healthcare services be concealed (14, 26).

Second, this study analyzes many socio-economic and expenditure variables together, including outsourcing. Proportion of region variation in amenable mortality attributed to this variable highlights that ensuring spending levels is not enough; attention must also be paid to the quality of publicly funded services and outsourced to private providers.

## Conclusion

In conclusion, this comprehensive analysis has highlighted that in decentralized systems performance, measuring in amenable mortality, can be different and it has unveiled several crucial determinants of amenable mortality and some noteworthy ones The positive association between the extent of healthcare outsourcing and amenable mortality serves as a potent reminder of the complexities inherent in healthcare system design and management. While the economic rationale behind outsourcing is evident, the potential repercussions on patient outcomes, as underscored by our findings, necessitate a more judicious approach.

The non-significance of per capita public and private health expenditure in the multivariate model suggests that aggregate spending levels alone may not adequately capture the organizational, allocative, and qualitative dimensions of healthcare delivery that influence amenable mortality. This finding should be interpreted cautiously, given the likely multicollinearity with socioeconomic variables and the short study period.

The negative association between household net income and healthcare system performance, supported by the positive association between the poverty rate and amenable mortality reinforces the intertwined nature of socioeconomic factors and health outcomes.

Policy makers should make an integrated reading of these findings to undertake measures capable of improving regional amenable mortality and should monitor healthcare system metrics, especially in the face of evolving practices such as outsourcing. It is important ensuring that economic imperatives do not overshadow the fundamental objective of healthcare: safeguarding patient wellbeing.

Unfortunately, limitations which are mostly related to data availability issues have not allowed us to consider other variables related to structure and organization of health care, such as the availability and qualifications of physicians, endowment of advanced medical equipment, number of hospital bed or existing medical procedures. These covariates as well, more results could be produced and may contribute to improved adjustment of health policies and efforts undertaken within the health care system at regional levels.

## Data Availability

Publicly available datasets were analyzed in this study. This data can be found here: https://demo.istat.it/ https://www.oecd.org/content/dam/oecd/en/data/datasets/oecd-health-statistics/avoidable-mortality-2019-joint-oecd-eurostat-list-preventable-treatable-causes-of-death.pdf, https://osservatoriosullasalute.it/osservasalute/rapporto-osservasalute-2020, https://www.salute.gov.it/new/it/tema/programmazione-e-finanziamento-del-ssn/dati-economico-finanziari-regionali/.
